# Long-Read Sequencing Emerging in Medical Genetics

**DOI:** 10.3389/fgene.2019.00426

**Published:** 2019-05-07

**Authors:** Tuomo Mantere, Simone Kersten, Alexander Hoischen

**Affiliations:** ^1^Department of Human Genetics, Radboud University Medical Center, Nijmegen, Netherlands; ^2^Laboratory of Cancer Genetics and Tumor Biology, Cancer and Translational Medicine Research Unit and Biocenter Oulu, University of Oulu, Oulu, Finland; ^3^Department of Internal Medicine, Center for Infectious Diseases (RCI), Radboud University Medical Center, Nijmegen, Netherlands; ^4^Radboud Institute for Molecular Life Sciences, Radboud University Medical Center, Nijmegen, Netherlands

**Keywords:** long-read sequencing, next-generation sequencing, medical genetics, structural variation, tandem repeat expansion, phasing, pseudogenes

## Abstract

The wide implementation of next-generation sequencing (NGS) technologies has revolutionized the field of medical genetics. However, the short read lengths of currently used sequencing approaches pose a limitation for the identification of structural variants, sequencing repetitive regions, phasing of alleles and distinguishing highly homologous genomic regions. These limitations may significantly contribute to the diagnostic gap in patients with genetic disorders who have undergone standard NGS, like whole exome or even genome sequencing. Now, the emerging long-read sequencing (LRS) technologies may offer improvements in the characterization of genetic variation and regions that are difficult to assess with the prevailing NGS approaches. LRS has so far mainly been used to investigate genetic disorders with previously known or strongly suspected disease loci. While these targeted approaches already show the potential of LRS, it remains to be seen whether LRS technologies can soon enable true whole genome sequencing routinely. Ultimately, this could allow the *de novo* assembly of individual whole genomes used as a generic test for genetic disorders. In this article, we summarize the current LRS-based research on human genetic disorders and discuss the potential of these technologies to facilitate the next major advancements in medical genetics.

## Introduction

Since its introduction over a decade ago, next-generation sequencing (NGS) of DNA has become a routine diagnostic tool for modern medical genetics and revolutionized the discovery of novel Mendelian disease genes (Gilissen et al., 2011). Due to the high-throughput nature and low costs of NGS compared to Sanger sequencing, previous single gene approaches have largely been replaced by gene panels and whole exome sequencing (WES), allowing better clinical diagnostics (Gilissen et al., 2011; Goodwin et al., 2016). However, despite the numerous advancements that NGS has conferred, many studies are still hindered by the short-read lengths (∼150–300 bp) that the current NGS technologies are bound to use in order to preserve high read quality (Goodwin et al., 2016). Issues arising from the use of short reads can mainly be pinned down to the highly repetitive and complex nature of the human genome (Feuk et al., 2006; de Koning et al., 2011; Treangen and Salzberg, 2011). It has been shown that despite the use of sophisticated bioinformatic algorithms it is often impossible to accurately map, or even assemble, short reads originating from regions harboring structural variation (SV), repetitive sequences, extreme guanine-cytosine (GC) content, or sequences with multiple homologous elements within the genome (Salzberg and Yorke, 2005; Treangen and Salzberg, 2011). This introduces errors in calling genetic variants and inability to capture certain genomic regions (Ashley, 2016). Furthermore, with short-read NGS (SR-NGS) the variant phasing information is often lost (Delaneau et al., 2013) and the data analysis is highly dependent on the reference genomes, which are known to be imperfect (Genovese et al., 2013). The dependency on the reference genome is especially problematic for the detection of SVs at complex genomic regions that can be highly individual- or population-specific (Chaisson et al., 2015b).

Genetics research has always been strongly driven by novel sequencing technologies, first by Sanger sequencing and later followed by NGS (Shendure et al., 2017). With the recently demonstrated success in identifying previously intractable DNA sequences and closing gaps in the human genome assemblies (Chaisson et al., 2015a; Seo et al., 2016; Shi et al., 2016; Jain et al., 2018), long-read sequencing (LRS) technologies hold the promise to overcome specific limitations of NGS-based investigations of human diseases. The main advantage of LRS compared to other platforms stems from the use of long-reads (>10 kilobase [kb] on average), originating from single DNA molecules (van Dijk et al., 2018). Moreover, the sequencing process occurs in real-time and both the sequencing and library preparation are conducted without the need of PCR amplification, therefore being free from any PCR related bias (Schadt et al., 2010). The absence of PCR leaves the DNA in its native state, enabling LRS technologies to directly detect base modifications, such as methylation (Flusberg et al., 2010; Laszlo et al., 2013).

**Figure 1:**
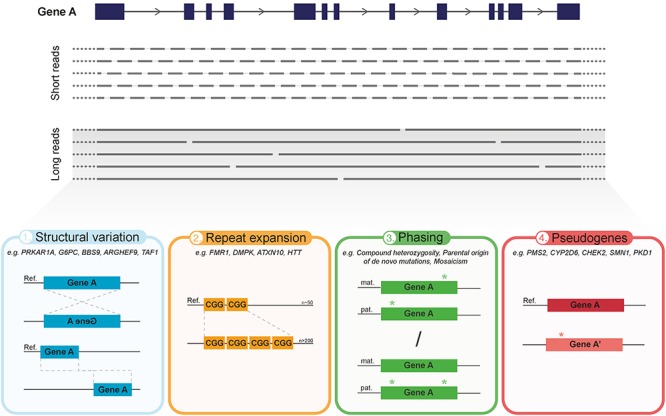
Overview of the main advantages of current long-read sequencing (LRS) approaches in medical genetics. The predominant difference between LRS and the conventional SR-NGS approaches is the significant increase in read length. In contrast to short reads (150–300 bp), LRS has the capacity to sequence on average over 10 kb in one single read, thereby requiring less reads to cover the same gene (illustrated in **top panel**). Hence, aside from reducing alignment and mapping errors, LRS holds various advantages over short-read approaches which can greatly impact medical genetics **(bottom panel)**. (1) Improved detection and characterization of large structural variation (SV), due to, e.g., large inversions or translocations. (2) Capacity to directly, and with that more accurately, sequence over long tandem repeat expansions and extreme GC-rich regions. (3) Enhanced phasing, i.e., assignment of genetic variants to the homologous paternal or maternal chromosomes, to determine inheritance patterns, parental origin of *de novo* events, mosaicism, allele specific expression and disease risk haplotypes. (4) Improved discrimination of clinically relevant genes from their pseudogenes.

Attributable to these features, LRS has the potential to grow into a technology that is used not only to produce high-quality genome assemblies (i.e., the platinum human reference genome) (Chaisson et al., 2015a; Pollard et al., 2018), but also to capture clinically relevant genomic elements which are problematic for conventional approaches (summarized in Figure 1). This has been shown in several studies by (1) identifying disease causing SVs that SR-NGS might not detect, (2) directly sequencing repeat expansions and regions with extreme GC-content, (3) resolving variant phasing and (4) distinguishing a gene of interest from its pseudogenes (Table 1). Importantly, recent studies have indicated that LRS technology may play an important role in discovering novel pathogenic mutations in human diseases with previously unknown genetic causes (Aneichyk et al., 2018; Ishiura et al., 2018; Zeng et al., 2018) and open up unprecedented opportunities to investigate transcriptomics (Byrne et al., 2017). In the future, this technology could ultimately allow whole genome sequencing (WGS) and even *de novo* assembly capturing all variant types present in individual genomes, independently of the reference genome limitations (Chaisson et al., 2015b). In this review, we summarize the LRS-based research of human genetic diseases and discuss the future promise of these technologies to enable the next major advancements in medical genetics. We focus on the prevailing true LRS methods that have been commercially released so far; single molecule real-time (SMRT) sequencing by Pacific Biosciences (PacBio) and nanopore sequencing by Oxford Nanopore Technologies Inc. (ONT) (Clarke et al., 2009; Eid et al., 2009) (Box 1 for technical summary).

**Table 1:** Human genetic diseases investigated with LRS technologies.

Phenotype	Technology	Finding	Reference(s)
**Identification and fine-mapping of structural variation**		
Developmental disorder	ONT	Complex rearrangements (chromothripsis)	Cretu Stancu et al., 2017
Carney complex	SMRT	Large deletion (*PRKAR1A*)	Merker et al., 2018
Bardet–Biedl syndrome	SMRT	Large deletion (*BBS9*)	Reiner et al., 2018
Glycogen storage disease IA	ONT	Large deletion (*G6PC*)	Miao et al., 2018
Developmental disorder	ONT	Chromosomal translocation	Dutta et al., 2018
X-linked Parkinsonism	SMRT and 10× genomics	SVA insertion (*TAF1*)	Aneichyk et al., 2018
**Sequencing over tandem repeat expansion loci**		
Fragile-X	SMRT	Repeat expansion length and interruption motifs (*FMR1*)	Loomis et al., 2013; Ardui et al., 2017; Ardui et al., 2018b
SCA10 and Parkinson’s disease	SMRT	Repeat expansion length and interruption motifs (*ATXN10*)	Schule et al., 2017; McFarland et al., 2015
ALS and FTD	SMRT and ONT	Repeat expansion length (*C9orf72*)	Ebbert et al., 2018
Huntington’s disease	SMRT	Repeat expansion length and somatic variability (*HTT*)	Hoijer et al., 2018
Myotonic dystrophy 1	SMRT	Repeat expansion length, interruption motifs and somatic variability (*DMPK*)	Cummings et al., 2017
BAFME and FCMTE	SMRT and ONT	Novel repeat expansion loci (*SAMD12*, *TNRC6A*, and *RAPGEF2*)	Ishiura et al., 2018; Zeng et al., 2018
Alzheimer’s disease	ONT	*ABCA7* repeat expansion length and alternative sequence motifs	De Roeck et al., 2017
**Resolving allele phasing**		
KID syndrome	SMRT	Revertant mosaicism (*CX26*)	Gudmundsson et al., 2017
Treacher Collins and Noonan syndrome	SMRT	Parental origin of *de novo* mutations (*TCOF1* and *PTPN11*)	Wilbe et al., 2017
**Discriminating pseudogenes**		
ADPKD	SMRT	Pseudogene discrimination (*PKD1*)	Borras et al., 2017
Primary immunodeficiency	SMRT	Pseudogene discrimination (*IKBKG*)	Frans et al., 2018
Drug metabolism	SMRT and ONT	Pseudogene discrimination and allele phasing (*CYP2D6*)	Ammar et al., 2015; Qiao et al., 2016; Buermans et al., 2017

*ADPKD, autosomal dominant polycystic kidney disease, ALS, amyotrophic lateral sclerosis; BAFME, benign adult familial myoclonic epilepsy; FCMTE, familial cortical myoclonic tremor with epilepsy; FTD, frontotemporal dementia; KID, Keratitis-ichthyosis-deafness; SCA10, spinocerebellar ataxia type 10; SVA, SINE-VNTR-Alu retrotransposon*.

**BOX 1. Technical summary of LRS technologies.**

**True versus synthetic LRS**

The current LRS technologies can be divided into true LRS and synthetic LRS technologies. In synthetic LRS approaches the long stretches of DNA are linked by molecular barcodes, which is followed by sequencing with conventional short-reads and *in silico* construction of the original DNA molecule (Goodwin et al., 2016). Usually these methods rely on serial-dilutions of very long DNA fragments and physical separation before library preparation in which molecule specific barcodes are added (Kitzman et al., 2011; Peters et al., 2012; Kuleshov et al., 2014). Similar technologies have recently been further optimized and commercialized by 10× Genomics Inc. (Weisenfeld et al., 2017). These technologies have already provided haplotype aware SR-WGS (Zheng et al., 2016) and improved SV calling from SR-WGS data (Nazaryan-Petersen et al., 2018; Marks et al., 2019). While synthetic long-reads leverage advantages of LRS, some short-read issues may persist, e.g., PCR-bias and intra-read complexity may not always be fully resolved.

**SMRT sequencing by PacBio**

As a first commercial platform for LRS, PacBio released the RS sequencer in 2011, which was followed by RSII in 2013 and Sequel in 2015. SMRT technology is based on special flow cells harboring individual picolitre-sized wells with transparent bottoms. Each of the wells, referred to as zero mode waveguides (ZMW), contain a single fixed polymerase at the bottom (Levene et al., 2003; Ardui et al., 2018a). This allows a single DNA molecule, which is circularized in the library preparation (i.e., the SMRTbell), to progress through the well as the polymerase incorporates labeled bases onto the template DNA. Incorporation of bases induces fluorescence that can be recorded in real-time through the transparent bottoms of the ZMW (Levene et al., 2003; Ardui et al., 2018a; Pollard et al., 2018). The average read length for SMRT was initially only ∼1.5 Kb, and with reported high error rate of ∼13% characterized by false insertions (Carneiro et al., 2012; Quail et al., 2012). However, the errors are randomly distributed across the reads (Eid et al., 2009) and high consensus sequences can be obtained with sufficient read depths (Roberts et al., 2013; Hebert et al., 2018). Also, for a single molecule, with ∼10 kb read length, each nucleotide position in a 1kb amplicon can be read ∼10 times using circular consensus sequence (CCS) method, rendering it unlikely that a same random mistake would occur in multiple reads (Travers et al., 2010; Hestand et al., 2016). Since its introduction, the read length and throughput of SMRT technology have substantially increased. Throughput can reach >10 Gb per SMRT cell for the Sequel machine, while the average read length for both RSII and Sequel is >10 kb with some reads spanning >100 kb (van Dijk et al., 2018). Still, the persisting downsides for this technology are the high cost/throughput ratio and the requirement of relatively high amounts of high quality DNA as starting material (Ardui et al., 2018a; van Dijk et al., 2018).

**Nanopore sequencing by ONT**

As an alternative, scientist have since many years aimed to use biological or synthetic nanopores to read the sequence of DNA molecules (Kasianowicz et al., 1996; Butler et al., 2008). In 2015, nanopore sequencing was commercially introduced by ONT with a portable MinION sequencer, which was followed by more high-throughput desktop sequencers GridION and PromethION. The basic principle of nanopore sequencing is to pass a single strand of DNA molecule through a nanopore which is inserted into a membrane, with an attached enzyme, serving as a biosensor (Deamer et al., 2016). Changes in electrical signal across the membrane are measured and amplified in order to determine the bases passing through the pore in real-time. The nanopore-linked enzyme, which can be either a polymerase or helicase, is bound tightly to the polynucleotide controlling its motion through the pore (Deamer et al., 2016; Pollard et al., 2018). For nanopore sequencing, there is no clear-cut limitation for read length, except the size of the analyzed DNA fragments. On average, ONT single molecule reads are >10 kb in length but can reach ultra-long for some individual reads lengths of>1 Mb surpassing SMRT (Jain et al., 2018). Also, the throughput per run of ONT GridION and PromethION sequencers are higher than for PacBio (up to 100 Gb and 6 Tb per run, respectively) (van Dijk et al., 2018). The raw reads have high error rates similar to SMRT dominated by false deletions and in particular homopolymer errors (Jain et al., 2015; Menegon et al., 2017). Currently, the errors are more systematic, and are related to the length and type of the DNA fragment in the nanopore itself, and thus may not all be overcome by just increasing the coverage as with SMRT sequencing (Krishnakumar et al., 2018).

**The main limitations of current LRS technologies**

**Library preparation:** for optimal LRS, fresh material or even intact cells are required. The DNA isolation protocols that are needed and the handling of ultra-long high molecular weight DNA require improvements.

**Error rate:** both LRS technologies still have a higher error rate compared to SR-NGS. SMRT sequencing may overcome this with CCS also for long-insert libraries (Wenger et al., 2019). It remains to be seen whether this is sufficient for all variant types including SNVs and indels. So far, ONT has more systematic error-profile, which may be more challenging to overcome.

**Costs:** while LRS is still more expensive than SR-NGS, recent advancements in throughput may offer even lower prices. ONT’s PromethION already offers 30× coverage WGS for less than 1,000 dollars, and PacBio’s 8M chip should also significantly reduce the price per human genome. It is also important to realize that the pricings are constantly changing in this rapidly developing field: https://docs.google.com/spreadsheets/d/1GMMfhyLK0-q8XkIo3YxlWaZA5vVMuhU1kg41g4xLkXc/htmlview?hl=en_GB (documentation of updated sequencing costs by Dr. A Vilella).

**Data analysis:** both raw data analysis as well as mapping and variant calling tools are much less mature for LRS than SR-NGS but are constantly being improved (Sedlazeck et al., 2018a).

## Overcoming Current Limitations in Medical Genetics

Whole exome sequencing (WES), mainly using Illumina platforms, is often used as a first-tier test for many genetic diseases. This has been very successful and has brought genetic testing and diagnostics into a whole new era (Gilissen et al., 2011). However, for many patients that have undergone diagnostic WES, or even WGS, the underlying cause of their disease remains unsolved (Gilissen et al., 2014). Curiously, the recent WGS studies applying LRS have revealed that each human genome harbors >20,000 of SVs (>50 bp) and additional thousands of indels (<50 bp), spanning dozens of megabases (Mbs), that have largely remained undetected with conventional SR-NGS (Chaisson et al., 2015b, 2019; Pendleton et al., 2015; Seo et al., 2016; Shi et al., 2016; Huddleston et al., 2017; De Coster et al., 2018). In patients with suspected genetic diseases, such hidden SVs and indels may well disrupt relevant genes or cause a dosage change for dosage-sensitive genes. Furthermore, NGS methods heavily rely on PCR, which leads to GC-content dependent coverage bias (Benjamini and Speed, 2012). Low or zero coverage at genomic locations with extreme GC-contents are estimated to span >150 Mb of gene-rich regions (Genomes Project Consortium et al., 2012) and some of those may as well account for a proportion of the hidden genetic variation underlying human diseases. Among the approaches that are based on SR-NGS, the PCR-free library preparation methods provide the most uniform genome coverage and may allow sequencing of some of the previously difficult regions (Dolzhenko et al., 2017). However, SR-NGS always requires a PCR-step in the template amplification, e.g., bridge-PCR, which may still introduce a sequencing bias.

In contrast to the contemporary SR-NGS methods, LRS technologies require no PCR amplification and sequencing is performed on single molecules instead of colonies or clusters of amplified DNA molecules, resulting in a more evenly distributed coverage (Schadt et al., 2010). This, together with longer reads that can span over challenging genomic regions and SVs, may offer an unprecedented view on previously poorly characterized genomic locations (Chaisson et al., 2015a; Jain et al., 2018) and eventually improve the variant calling of population-scale sequencing data (Ameur et al., 2018a). While increases in the throughput and lower prices may in the future allow more long-read WGS for individual patients, targeted approaches are already demonstrating the value of LRS for medical genetics (Tables 1, 2) (Ameur et al., 2018b).

**Table 2:** Different applications of LRS technology.

LR-WGS	
SMRT-WGS	*De novo* assembly and reference-based WGS with focus on structural variant calling (Chaisson et al., 2015a; Seo et al., 2016; Shi et al., 2016).
ONT-WGS	*De novo* assembly and reference-based WGS utilizing ultra-long reads to improve phasing and close gaps in the reference genome (Jain et al., 2018).
**Targeted LRS**	
LR-PCR amplicon sequencing	Commonly used targeted approach with standard LR-PCR amplification of the target region followed by SMRT amplicon sequencing (Borras et al., 2017; Frans et al., 2018)
Hybridization-based capture	As for SR-NGS, hybridization-based target capture can be applied for LRS (Wang et al., 2015). Protocols are available for different vendors of bait-libraries.
No-Amp targeted SMRT sequencing	A standard PacBio SMRTbell library is created and a Cas9 guide RNA is designed adjacent to the region of interest. Digestion with Cas9 breaks open the SMRTbell molecules to enable ligation with a capture adapter. SMRTbell molecules that contain the capture adapter are enriched on magnetic beads and prepared for SMRT Sequencing (Tsai et al., 2017; Hoijer et al., 2018).
CATCH for ONT sequencing	CATCH (Cas9-assisted targeting of chromosome segments) is based on targeted fragmentation of DNA *in vitro* by Cas9, followed by separation of the target region from the rest of the genomic DNA by pulsed field gel electrophoresis and DNA isolation from the gel (Jiang et al., 2015; Gabrieli et al., 2018)
ONT Read until selective sequencing	Real-time data analysis that enables the selection of specific DNA molecules for sequencing by reversing the driving voltage across individual nanopores: this enables to proceed to sequence only molecules that are recognized to originate from a certain chromosome or region of interest (Loose et al., 2016).
**LR-RNA-sequencing**	
SMRT-IsoSeq	The IsoSeq method of PacBio enables sequencing of full-length transcripts up to 15 Kb using SMRT sequencing, in turn eliminating computational transcript reconstruction and the need for a reference genome.
Direct ONT RNA-seq	By circumventing the bias prone elements in regular RNA sequencing, i.e., reverse transcription and PCR amplification of cDNA, Nanopore’s direct RNA-seq enables the direct detection of full-length RNA. This real-time single-molecule method is based on two adapters; (1) a poly(T)adaptor for recognition and binding of the polyadenylated messenger RNA, and (2) a pair of sequencing adaptors that ligate onto the overhang of the poly(T)adaptors and facilitate its capture by a nanopore (Garalde et al., 2018).
R2C2 method for ONT	Rolling Circle Amplification to Concatemeric Consensus (R2C2) method enables to generate a consensus from a single sequence read with many copies of an original molecule: this approach has been used to accurately produce full-length RNA transcript isoforms (Volden et al., 2018).

### Detection and Characterization of Structural Variation

High-depth sequencing by NGS is a reliable and cost-efficient method for detecting single nucleotide variants (SNVs) and small indels (Ashley, 2016; Goodwin et al., 2016). However, in the detection of larger genomic deletions, duplications and rearrangements, SR-NGS approaches often lack sensitivity, show excess of false positives and misinterpret complex SVs (Tattini et al., 2015; Ashley, 2016; Huddleston et al., 2017; Sedlazeck et al., 2018b). Yet, SVs >50 bp in size are an important source of genetic variation, accounting for the greatest number of divergent bases across human genomes (Alkan et al., 2011; Sudmant et al., 2015; Chaisson et al., 2019). Moreover, SMRT sequencing of a haploid human genome has shown that as much as 89% of the identified variation, consisting mainly of SVs, had been missed in the 1000 genomes project (Huddleston et al., 2017). Similarly, a sevenfold increase in SV detection was achieved by a multi-platform approach, including LRS, compared to standard SR-NGS methods (Chaisson et al., 2019). It is likely that this gain in SV detection sensitivity is due to the different sequencing technology itself, as this benefits from longer fragments, no PCR, and less sequence bias.

The detection of SVs by SR-NGS is indirect and based on read depth coverages and use of paired-end reads, for which the approximate length between read pairs is known (Tattini et al., 2015). For copy number variants (CNVs), the excess of reads can indicate amplification whereas the loss of reads is suggestive for a deletion. Rearrangements can be discovered using paired-end reads by investigating deviations in the expected distances and orientations between read pairs (Tattini et al., 2015). In contrast to SR-NGS, the long reads obtained with PacBio or ONT sequencers are more likely to span SV breakpoints, or often the entire SV event, with high-confidence alignments (Huddleston et al., 2017; De Coster et al., 2018; Sedlazeck et al., 2018b). Furthermore, longer reads can be more confidently aligned to repetitive sequences that often mediate the formation of SVs (Sedlazeck et al., 2018b). Long reads also allow better distinction of haplotypes, further contributing to the accuracy of analyzing SVs (Seo et al., 2016; Cretu Stancu et al., 2017). Other methods, such as microarray copy number profiling and karyotyping, have traditionally been used to detect disease causing SVs. However, with these approaches it is not possible to map small or copy-balanced SVs, and they do not provide accuracy at base-pair resolution, nor the possibility to resolve complex SVs (Alkan et al., 2011). Thus, LRS can substantially increase the resolution and reliability of SV detection and mapping.

Recently, Cretu Stancu et al. (2017) proposed LRS as an alternative approach for genome-wide detection of clinically relevant SVs and used ONT-WGS to analyze two patients with congenital abnormalities caused by *de novo* chromothripsis rearrangements. The authors demonstrated that long reads, even with a relatively low coverage of 16×, are superior to short reads (WGS with average coverage of ∼30×) in detecting and mapping the breakpoints of the rearrangements. The improved phasing capability with long reads also enabled the determination of the parental origin of these *de novo* events (Cretu Stancu et al., 2017). Other studies have demonstrated the potential benefits of LRS over standard SR-NGS in clinical diagnostics to detect pathogenic SVs. In a study by Merker et al. (2018), a patient with clinically diagnosed Carney complex had previously received negative test results from diagnostic gene panel sequencing and WGS. In contrast, low-coverage SMRT-WGS successfully identified the causative heterozygous deletion of over 2 kb overlapping the first exon of *PRKAR1A* (Merker et al., 2018). Similarly, Miao et al. (2018) explored ONT-WGS to confirm the diagnosis for a patient with recessive glycogen storage disease by identifying a second-hit (∼7 kb deletion) in *G6PC*, which had remained undetected in previous WES analysis. Together, these studies indicate that LRS has the ability to surpass conventional NGS-methods in the detection of SVs and yield clinical diagnoses at a molecular level in previously unsolved cases.

The fine-mapping of SVs at single-nucleotide resolution is important for patients in which translocation or inversion breakpoints disrupt disease causing genes (Chen et al., 2010). For this, long reads are highly beneficial and may render mapping by laborious amplicon-based Sanger sequencing obsolete, as demonstrated by Reiner et al. (2018) who used SMRT to fine-map *BBS9* deletion in a patient with Bardet–Biedl syndrome. In another diagnostic case-study, ONT-WGS was able to track down the exact breakpoints of a reciprocal translocation, which led to the identification of disrupted *ARHGEF9* gene in a girl with intellectual disability (Dutta et al., 2018).

In addition to the improved identification and mapping of SVs affecting known disease genes, we are now seeing the first discoveries of novel disease genes harboring SVs that are characterized with LRS technologies. As an outstanding example, an unbiased *de novo* assembly using LRS (SMRT and synthetic longs reads with 10× Genomics) was important for the identification of SINE-VNTR-Alu insertion in *TAF1*, a newly discovered gene for X-linked Dystonia Parkinsonism (Aneichyk et al., 2018). While multiple studies indicate that LRS is superior to SR-NGS in the discovery of SVs, it is also shown that once the alternate allele is resolved, many of these events (∼61%) can be genotyped with high accuracy and lower costs using SR-NGS (Huddleston et al., 2017). This shows that part of the missed SVs are present in the SR-NGS data and therefore improved software may still provide a more complete picture of genomes sequenced with short reads. It is noted that the current cut-off of 50 bp for SVs is arbitrary; indels <50 bp are also identified by LRS and their frequency in WGS is even much higher than for the >50 bp SVs (Chaisson et al., 2019). In addition to PacBio and ONT, synthetic LRS (i.e., by 10× Genomics) can be highly useful in SV detection (Nazaryan-Petersen et al., 2018; Marks et al., 2019). Uncovering SVs and indels comprehensively holds the potential to unravel a number of novel disease causing mutations underlying human diseases.

### Sequencing Tandem Repeat Expansions

A short tandem repeat is a region of genomic DNA with multiple adjacent copies of short (1–6 bp) sequence units. These repeat regions are highly mutable due to replication errors that occur during cell divisions and to date over 30 human diseases known to be caused by tandem repeat expansions (Tang et al., 2017) or repeat contractions (Lemmers et al., 2018). Since their discovery, the research of tandem repeat expansions has been limited by the overall incompliance of these genomic elements to standard molecular techniques like cloning, PCR and sequencing (Loomis et al., 2013). Most of the disease causing expansions are longer than the currently used NGS reads, making it virtually impossible to accurately assemble those (Treangen and Salzberg, 2011; Lee et al., 2016; Sedlazeck et al., 2018a). Multiple studies have now shown that LRS technologies are well suited to transcend through these long, often GC-rich, repeat expansions (Table 1). This not only allows the direct detection of expansion lengths, but also the intra-molecule sequence variation, which might provide clinically relevant additional information (Ardui et al., 2018b; Cumming et al., 2018; Hoijer et al., 2018; McFarland et al., 2015).

The first tandem repeat associated disease studied by LRS was Fragile-X (Loomis et al., 2013), which is caused by CGG-repeat expansion in the 5′UTR of the *FMR1* gene (Kremer et al., 1991). With standard DNA sequencing technologies, i.e., SR-NGS and Sanger sequencing, it has been impossible to directly sequence the expanded CGG-repeats, consisting of >200 units at full mutation range (Nolin et al., 2003; Peprah, 2012). SMRT technology enabled for the first time to completely sequence expanded full mutation *FMR1* alleles, up to 750 CGG-repeats, which translates to >2 kb of 100% CGG-repeat DNA (Loomis et al., 2013). The obtained sequencing data and phasing information further allowed to define the presence of ‘AGG’ interruptions, which affect the risk of a premutation to expand into a full mutation in the following generation (Nolin et al., 2015; Ardui et al., 2017). The clinical use of this information has been largely neglected due to technical limitations, which can now be overcome with LRS (Ardui et al., 2018b). Genetic variation within repeat expansion loci that are causative for other diseases, such as myotonic dystrophy 1 (DM1) and spinocerebellar ataxia 10 (SCA10), have also been investigated with LRS technology (Cumming et al., 2018; McFarland et al., 2015). In DM1, SMRT sequencing detected *de novo* repeat interruptions at *DMPK* expansion locus, associated with reduced somatic instability of the repeat expansion and therefore mild or even absent clinical features (Cumming et al., 2018). Moreover, McFarland et al. (2015) demonstrated that SMRT sequencing could identify interruption motifs, potentially acting as phenotypic modifiers, within the tandem repeat expansion locus in *ATXN10* of SCA10 patients.

The presented studies on repeat expansions rely on PCR-based target enrichment, which may complicate the analysis due to the occurrence of PCR stutter, chimeric molecules, and false insertions or deletions of the repeat units (Laver et al., 2016; Tsai et al., 2017; Hoijer et al., 2018). Therefore, it has been proposed that the optimal approach to study these repetitive regions would be to sequence single DNA molecules without any prior PCR amplification (Hoijer et al., 2018). For this, Tsai et al. (2017) have developed CRISPR/Cas9-based amplification-free target enrichment method (No-Amp targeted sequencing, Table 2), which was used to capture repeat the expansion locus in Huntingtin (*HTT*), responsible for Huntington’s disease (Hoijer et al., 2018). This allowed the retrieval of detailed sequence information and assessment of somatic variability of repeat elements without the interference of PCR stutter. In addition, No-Amp approach has been utilized to successfully sequence across *C9orf72* and *ATXN10* repeat expansion loci in patients with frontotemporal dementia or Parkinson’s disease, respectively (Schule et al., 2017; Ebbert et al., 2018). These studies indicate that No-Amp Targeted sequencing could provide an optimal targeted solution to study different repeat elements that are recalcitrant to PCR-based methods.

In the era of CRISPR/Cas9 genome editing, LRS has found use in monitoring the efficiency of editing challenging genomic regions. Dastidar et al. (2018) excised a disease causing tandem repeat expansion in the *DMPK* locus in DM1 patient-derived cell lines and used SMRT sequencing to monitor the efficiency of the excision. In the future, LRS could be more broadly implemented in genome editing of challenging regions. Moreover, with the capability of directly sequencing through long tandem repeats, LRS technology is already accelerating the discovery and characterization of novel repeat expansions linked to human diseases. So far, this has been demonstrated in familial forms of epilepsy, bipolar disorder and schizophrenia (Ishiura et al., 2018; Song et al., 2018; Zeng et al., 2018). It is noted that, while the current approaches still largely rely on targeted enrichment of suspected repeat expansion loci, ultimately the analysis of all repeat-expansions genome-wide will be retrieved directly by WGS with long reads (De Roeck et al., 2018; Chaisson et al., 2019).

### Haplotype Resolution With Long Reads

Phasing refers to the means of assigning genetic variants to the homologous paternal and maternal chromosomes. In medical genetics, phasing is very important for understanding, e.g., inheritance patterns, parental origin of *de novo* mutations, mosaicism, allele-specific expression and disease risk-haplotypes (Tewhey et al., 2011). However, in order to directly resolve the haplotype of two heterozygous SNVs they would need to be covered by the same molecule (i.e., read). With SR-NGS this is usually only the case for a limited number of variants (20–30% of substitutions), despite the use of paired-end reads (Goldmann et al., 2016). Therefore, phasing has largely been based on parental genotypes and statistical imputation (Tewhey et al., 2011) or, in some cases, laborious physical separation of entire chromosomes (Fan et al., 2011). Now, with the substantially longer read lengths, LRS technology enables to directly phase variants multiple kbs apart (Laver et al., 2016) or even assemble and phase complex genomic regions, such as the major histocompatibility complex, in full-length in a diploid human genome (Jain et al., 2018).

Knowing whether two variants in the same gene are in *cis* or *trans* can be crucial for diagnostic purposes, especially for compound heterozygosity, which is a commonly observed phenomenon underlying recessive Mendelian disorders (Tewhey et al., 2011). By using LRS, two SNVs in the same gene can be directly phased without the need of testing the parents (Laver et al., 2016) or if one of the two mutations has occurred *de novo* and the parental genotypes cannot resolve the phase in the offspring (Neveling et al., 2012). In pre-implantation genetic diagnostics (PGD), LRS can be applied to determine the parental origin of pathogenic *de novo* mutations by simply sequencing across the adjacent variants (Wilbe et al., 2017). Resolving the parental origin of *de novo* mutations is needed for estimating the recurrence risk of a genetic disease in the case of germline mosaicism (Dimitriadou et al., 2017). LRS could also be used in PGD to directly phase parental genotypes for genome-wide haplotype reconstruction of a single cell via discrete SNP genotypes (i.e., haplarithmisis) (Zamani Esteki et al., 2015). Moreover, phasing with long reads offers an accurate method to study mosaicism. This has recently been shown by Gudmundsson et al. (2017) who used SMRT to resolve allele phasing in an exceptional case of keratitis-ichthyosis-deafness syndrome exhibiting revertant mosaicism, a rare phenomenon involving spontaneous correction of a pathogenic mutation by another mutation in a somatic cell. To keep in mind, Laver et al. (2016) pointed out problems in resolving allele phasing with targeted SMRT amplicon sequencing. In their study, the formation of chimeric molecules during PCR-amplification led to unambiguous results, which could have been avoided by using PCR-free methods (Ebbert et al., 2018; Hoijer et al., 2018). However, these methods can be costly and time consuming and simply keeping the amount of PCR cycles as low as possible could offer an easier solution (Laver et al., 2016).

Identification of SVs may also benefit from phasing (Seo et al., 2016; Cretu Stancu et al., 2017), in particular SV calling in cancer genomes can be significantly improved to understand the complexity of chromosomal rearrangements (Nattestad et al., 2018). Furthermore, phasing with LRS can be used to reveal the subclonal heterogeneity in malignancies (Lode et al., 2018). For genome-wide association studies (GWAS), multi-allelic haplotype markers can provide superior power compared to single-SNP markers in mapping disease loci (Akey et al., 2001; Khankhanian et al., 2015). However, the implementation of haplotype-based GWAS is highly dependent on capability to phase alleles (He et al., 2011). Therefore, LRS could be utilized to fine-map genetic associations within regions of interest that have previously been identified by GWAS. In addition to these multiple situations where accurate phasing is of great importance, LRS technology and the emerging ultra-long reads (>100 kb) from latest LRS developments may eventually produce fully phased diploid genomes (Pendleton et al., 2015; Seo et al., 2016; Jain et al., 2018; Chaisson et al., 2015b), which can even originate from single cells (Hills et al., 2018). This would allow for research that fully leverages phase information in order to understand how phenotypes are influenced by unique haplotype combinations (Tewhey et al., 2011).

### Pseudogenes

Pseudogenes usually originate from gene duplication or retrotransposition events and are defined as sequences that resemble known functional genes but cannot produce functional proteins (D’Errico et al., 2004). Notably, the GENCODE project has estimated that the human genome contains more than 14,000 pseudogenes (Pei et al., 2012) and many clinically relevant genes (such as *PMS2, CYP2D6, CHEK2, SMN1*, and *PKD1*) are known to have pseudogenes with high sequence homology (Mandelker et al., 2016). This has significant consequences for re-sequencing studies as pseudogenes may severely impair reliable variant identification in their functional counterparts (Claes and De Leeneer, 2014) and lead to false diagnostic test results (Bardaro et al., 2003). Traditional methods, such as SR-NGS may capture sequences from pseudogenes in addition to the functional genes, leading to mapping errors, low variant detection rates and high number of false positives (Claes and De Leeneer, 2014). Other issues originate from the fact that some pseudogene sequences are still missing from the human reference genome and may cause mapping issues for SR-NGS (Karakoc et al., 2011). Therefore, assays based on SR-NGS may not always be reliable for investigating genes that have highly homologous pseudogenes.

To date, several studies (Table 1) have utilized LRS in order to investigate clinically relevant genes with homologous pseudogenes (Ammar et al., 2015; Qiao et al., 2016; Borras et al., 2017; Buermans et al., 2017; Frans et al., 2018). These studies have largely relied on target enrichment with long-range PCR (LR-PCR) using primers that locate to the rare mismatch sites that distinguish the gene of interest from its pseudogenes (Frans et al., 2018). Similar approaches have been used with Sanger sequencing and SR-NGS. However, with LRS, the reads can span the complete LR-PCR amplicon and retain the phase information (Buermans et al., 2017). Furthermore, reference-free assembly of long reads can circumvent errors that may be introduced by complex or repetitive regions in the reference genome (Borras et al., 2017). For example, Polycystin 1 (*PKD1*), responsible for autosomal dominant polycystic kidney disease, has traditionally been challenging to analyze with NGS or Sanger sequencing because of its high GC-content, large size and homology with pseudogenes (Tan et al., 2014). Borras et al. (2017) demonstrated that by coupling LR-PCR with SMRT, the interference of residual *PKD1* pseudogene amplification and alignment ambiguities could be eliminated, and SVs could be identified. In addition, multiple studies have demonstrated the utility of LRS for genotyping and phasing *CYP2D6*, which is one of the most important genes involved in drug metabolism, but has been difficult to analyze by conventional methods due to homologous pseudogenes and complex gene rearrangements (Ammar et al., 2015; Qiao et al., 2016; Seo et al., 2016; Buermans et al., 2017).

For medical genetics research, it is crucial to have reliable information on the population frequencies of particular gene variants in order to assess their potential pathogenicity (Ashley, 2016). For this, databases such as ExAC and GnomAD are often used (Lek et al., 2016). However, reference datasets attained with SR-NGS might be problematic for genes with highly homologous pseudogenes. This is mainly because: (1) the deposited variants may be derived from pseudogenes, (2) true variants might stay undetected or (3) the gene of interest might be ambiguously covered (Mandelker et al., 2016). Long reads could be used to improve the annotation of duplicated regions because they harbor sufficient number of paralogous sequence variants to confidently assign them to their respective paralogs (Dougherty et al., 2018). Therefore, in addition to more reliable pseudogene discrimination, LRS can be used to generate more accurate reference datasets and annotations for many challenging genes, subsequently allowing improvements in variant interpretation.

## Uncovering the Transcriptome Landscape to Aid Genetic Diagnosis

The emergence of NGS, together with the incessancy of technological advances, drives our increasing ability to uncover complex and previously ‘hidden’ genetic aberrations, however, our current capacity to interpret their functional and clinical impact remains restrained. By providing a global transcriptomic profile, both quantitatively, i.e., gene expression levels, and qualitatively, i.e., transcript sequences and isoform structures, RNA sequencing holds the potential to reduce this diagnostic gap significantly. Recently two research groups pioneered this technology as a complementary tool to DNA-based tests and demonstrated its value in the detection of both coding and non-coding variants (Cummings et al., 2017; Kremer et al., 2017). In particular, authors identified a variety of variants underpinning (1) abnormal or mono-allelic expression and (2) aberrant splicing, resulting in the creation, skipping and truncation of exons, as well as exon and intron retention, even in regions harbouring repetitive regions. In turn, resulting in a diagnostic yield ranging from 10% for mitochondriopathies to 21% for primary muscle disorders in patients lacking strong candidates from WES or WGS (Cummings et al., 2017; Kremer et al., 2017).

**Figure 2:**
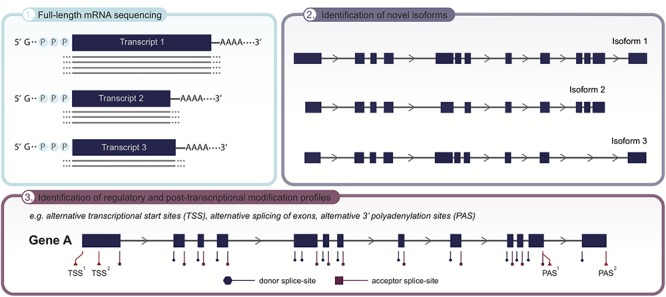
Applicability of long-read sequencing (LRS) to unveil the transcriptome landscape of cells and tissues. Given the significant improvements in read length, employing LRS on RNA level now allows for full-length isoform sequencing, covering the complete mRNA transcript in one single read **(panel 1)**. As recent advances have demonstrated the isoform landscape to be more complex than initially thought, LRS holds the potential to identify novel isoforms **(panel 2)**, as well as detect transcriptional and post-transcriptional modification sites, e.g., alternative transcriptional start sites (TSS), alternative splicing of exons and alternative transcription termination sites (3′polyadenylation sites; PAS), that underpin the emergence of different isoforms **(panel 3)**. Collectively, uncovering the full isoform diversity within cells and tissues.

Similar to NGS-based genomic approaches, however, short-read RNA sequencing methodologies are limited by their need for computational reconstruction of individual short reads into complete transcripts (Steijger et al., 2013). With the advent of long-read RNA sequencing the accurate identification and quantification of full-length mRNA transcript isoforms has become possible (Figure 2). A comparative approach by Anvar et al. (2018) has revealed up to 17% of novel alternative exons to be detected following long-read RNA sequencing of three primary human tissues, i.e., brain, heart, liver, thereby corroborating the isoform landscape to be more complex than initially thought (Sharon et al., 2013; Tilgner et al., 2013). In addition, the authors demonstrate the applicability of full-length mRNA sequencing to uncover transcriptional regulation and post-translational modification profiles, including alternative transcription initiation, allele specific alternative splicing and alternative 3′ termination (polyadenylation) (Anvar et al., 2018). The clinical significance of integrating this transcriptomic data was exemplified by Aneichyk et al. (2018), who identified an intronic retrotransposon to induce alternative splicing that subsequently affects *TAF1* expression, in turn causing X-linked Dystonia-Parkinsonism. Moreover, by exploring long-read RNA sequencing in the characterization of premature termination codons in *ABCA7* in late-onset Alzheimer’s disease, De Roeck et al. (2017) observed various degrees of nonsense-mediated decay (NMD) and transcript modification that potentially influence *ABCA7* dosage and disease severity. While the ultimate goal may be to employ this technique to allocate the causative variant and map its functional impact on a patient-level, there may already be a major improvement by sequencing reference sets of tissue/cell specific RNA-isoform landscapes. Gaining insight into the isoform landscape of disease relevant tissues or cell types, potentially even on a single-cell level (Byrne et al., 2017; Gupta et al., 2018), will advance the overall interpretation of clinical significance of complex rearrangements (Nattestad et al., 2018), tissue-specific isoforms (Clark et al., 2018) and splice variants (de Jong et al., 2017). Ultimately, this knowledge can be implemented to improve WES/WGS-based variant filtering, prioritization and prediction of their functional impact.

## Future Perspectives and Conclusion

Most of the clinically relevant examples described in this review use targeted LRS approaches, indicating that the broader use of LRS could significantly increase the diagnostic yield of genetic testing and discover novel disease genes. Especially SVs, repetitive elements and complex genomic regions that are difficult to assess with short reads can now be better assessed. LRS technology is also changing our view on the mRNA isoform landscape of different tissues and genes, potentially enabling better functional interpretation of genomic variation in the future. It is noted that, while the currently used LRS technologies are not quite yet at the stage of individual fully phased *de novo* assembled genomes, but with increased throughput, higher accuracy and lower costs, LRS-based WGS is coming within reach. Once this is routine, we are foreseeing the possibility that WGS with long reads could serve as a truly generic test that enables to detect all genetic variants present in an individual’s genome. Then the power of LRS to overcome the current limitations in medical genetics may be elucidated at fast pace. Moreover, systematic studies utilizing LRS in patient cohorts with unsolved genetic diseases and control populations are warranted, and several consortia, e.g., www.solve-rd.eu or http://www.internationalgenome.org/1000-genomes-browsers, have already announced the use of LRS-WGS for this.

While the medical genetics community is now starting to realize the potential of this technology, some obstacles need to be overcome in order for LRS to be established as a mainstream tool (for overview of LRS limitations, see Box 1). Especially library preparation and analysis still need to reach the high level of robustness of SR-NGS technologies. For the data analysis, bioinformatic tools have traditionally been optimized for short-read sequencing data and need to be adapted, as well as thoroughly tested, for long reads (Sedlazeck et al., 2018a). In addition, analyzing whole genomes with LRS is still very expensive and the future application will depend largely on pricing progress of all NGS technologies, and may also depend on how fast the short-reads can reach an even lower prices with even better quality (i.e., the ∼100$ genome). Eventually, one might need to consider a choice between sequencing many less-expensive genomes with short-reads as opposed to investigating a smaller number of genomes in-detail using long reads. It is not a straightforward decision to make, however, the latter option would ideally be beneficial for an individual patient in clinical diagnostics. Combining SR-NGS and LRS can also be a powerful approach for highly accurate variant calling and assembly (Chaisson et al., 2019) and become increasingly important and more easily available with the planned acquisition of PacBio by Illumina.

We foresee that both of the prevailing LRS technologies, PacBio and ONT, will have a major influence on the future of medical genetics. ONT has the potential to become low in price, ease of use technology that is capable of directly investigating both native DNA and RNA in a high-throughput manner. The main challenge for ONT technology is to circumvent the systematic error rate, e.g., for homopolymer regions, which may be a limitation especially for future diagnostic applications. ONT has put forward ideas to improve this by optimizing bioinformatic tools that allow more accurate repeat lengths measurements and proposed to leverage different types of nanopores. One possible improvement was already enabled by the rolling circle amplification to concatemeric consensus method (R2C2) (Volden et al., 2018). For SMRT sequencing, the consensus accuracy achieved is better, especially once intra-molecule consensus – in addition to the existing inter-molecule consensus – calling is also feasible for long-insert libraries. To enable human genome sequencing, throughput needs to go up. To this end, PacBio has recently announced to release a SMRT cell with 8 million ZMWs in 2019 (∼8-fold increase) and other improvements may come from even longer read lengths. This would enable much higher throughput and reduction in sequencing costs. Altogether, it remains to be seen whether the quality of the LRS technologies is sufficient for genome-wide detection of small indels and SNVs. If so, PacBio and ONT will have a chance to become the platform for true WGS for population scale studies in the near future. Moreover, the native epigenetic modifications of the DNA are preserved in PCR-free single molecule sequencing, opening up possibilities for novel LRS-based applications to assess base modifications (Pham et al., 2016; Euskirchen et al., 2017).

In addition to already commercially available technologies discussed in this review, others are in active development and will hopefully be available for users in the future. These include novel nanopore-based technologies from Genia (now acquired by Roche) and Stratos Genomics (Carson and Wanunu, 2015; Fuller et al., 2016), or the Electronic Nano-Device sequencing (ENDSeq) from Roswell Biotechnologies (Pugliese et al., 2015). Other exciting technologies that may also provide long-read insights are, e.g., library-and amplification-free technology from Nanostring (Hyb & Seq), nanochannel genome mapping technology from Bionano Genomics (Lam et al., 2012; Seo et al., 2016) and Strand-Seq that preserves long-range context of homologous chromosomes (Sanders et al., 2017; Ghareghani et al., 2018). Whether these or other technologies are complementary to the existing LRS approaches, or may be able to compete with or even replace PacBio and ONT remains to be seen.

To conclude, we are on the advent of next revolution in sequencing technology. Once the major obstacles regarding accuracy, analysis and pricing are overcome, the time would be right to move towards individual fully phased long-read genomes, likely based on individual *de novo* assemblies that enable the identification of all the genetic variants regardless of their type.

## Author Contributions

AH and TM conceived the study. TM, SK, and AH drafted manuscript. SK prepared the figures.

## Conflict of Interest Statement

The authors declare that the research was conducted in the absence of any commercial or financial relationships that could be construed as a potential conflict of interest.
